# To Prevent Gangrene from Cold

**Published:** 1856-02

**Authors:** 


*To prevent Gangrene from Cold.*

A writer in the *London Lancet* calls attention to the fact that the Russian peasantry often rub their feet with tallow and oil to prevent the effects of cold and the statement of a French surgeon that the soldiers who rubbed their noses and ears with

*goose fat* were not frost-bitten. The writer had observed, that birds much exposed to cold, had a layer of fat under the skin. He gives this advice in regard to the feet, for those that are to be much exposed to intense cold—" Rub the feet with fat or oil, then cover with wash-leather socks, and over these the ordinary worsted or woolen socks," to which we would add thick leather boots, well saturated with tallow.

